# Malondialdehyde as a Potential Oxidative Stress Marker for Allergy-Oriented Diseases: An Update

**DOI:** 10.3390/molecules28165979

**Published:** 2023-08-09

**Authors:** Raffaele Cordiano, Mario Di Gioacchino, Rocco Mangifesta, Claudia Panzera, Sebastiano Gangemi, Paola Lucia Minciullo

**Affiliations:** 1 Unit and School of Allergy and Clinical Immunology, Department of Clinical and Experimental Medicine, University of Messina, 98125 Messina, Italy; raffaelecordiano@gmail.com (R.C.); claudiapanzera07@gmail.com (C.P.); sebastiano.gangemi@unime.it (S.G.); paolalucia.minciullo@unime.it (P.L.M.); 2 Center of Advanced Science and Technology (CAST), G. D’Annunzio University, 66100 Chieti, Italy; rocco.mangifesta@unich.it; 3 YDA—Institute of Clinical Immunotherapy and Advanced Biological Treatments, 65121 Pescara, Italy

**Keywords:** malondialdehyde, oxidative stress, inflammation, allergy, rhinitis, asthma, urticaria, atopic dermatitis

## Abstract

Malondialdehyde (MDA) is a compound that is derived from the peroxidation of polyunsaturated fatty acids. It has been used as a biomarker to measure oxidative stress in various biological samples in patients who are affected by a wide range of diseases. The aim of our work is to provide an updated overview of the role of MDA as a marker of oxidative stress in allergy-related diseases. We considered studies involving both paediatric and adult patients affected by rhinitis, asthma, urticaria and atopic dermatitis. The measurement of MDA was performed on different types of samples. The reported data highlight the role of serum MDA in inflammatory airway diseases. According to the literature review, the oxidative stress status in asthmatic patients, assessed via MDA determination, appears to worsen in the presence of other allergic airway diseases and in relation to the disease severity. This suggests that MDA can be a suitable marker for monitoring the disease status. However, there are several limitations in the considered studies due to the different samples used and the lack of phenotyping and description of the clinical period of patients examined. In cutaneous allergic diseases, the role of MDA is controversial because of the smallness of the studies and the heterogeneity of the samples and patients.

## 1. Introduction

### 1.1. Generalities

Oxidative stress is defined as an imbalance between antioxidants and pro-oxidants, favouring the latter, and is recognised as a key mechanism that impairs molecular signalling pathways and enzyme activities, leading to tissue damage [1]. Reactive oxygen species (ROS) are the primary effector molecules of oxidative stress, which are produced under physiological states, such as during cell metabolism, and under pathological conditions. Endogenous sources of ROS include the mitochondria, plasma membrane, endoplasmic reticulum and peroxisomes, where enzymatic reactions and the autoxidation of various compounds occur [2]. Exogenous factors, such as UV exposure, chronic stress, intense exercise, infections, allergens and pollutants, also contribute to ROS production [3,4].

Reactive species play a dual role in cellular homeostasis, exerting both beneficial and harmful effects. They are involved in the physiological cellular responses to pathogenic stimuli, activating immune cells like neutrophils, macrophages and T lymphocytes. They elicit both mitogenic and pro-apoptotic responses and contribute to the regulation of numerous cellular signalling pathways, particularly the JNK and p38 MAPK-related cascades [5,6,7]. Their detrimental effects include damage to nucleic acids, proteins and lipids [8]. As the oxidative state increases, the enzymatic and non-enzymatic antioxidant systems enhance their activities to counteract cellular stress. The key enzymes and antioxidants that are involved in the line of defence against oxidative stress include glutathione peroxidase (GSX/Px), superoxide dismutase (SOD), catalase (CAT), glutathione (GSH), carotenoids, flavonoids, ascorbic acid and alpha tocopherol [9].

When the extent of oxidative damage exceeds the capacity for repair, cellular damage occurs. An impaired redox balance has also been linked to numerous diseases, the ageing process and carcinogenesis [10,11,12]. Moreover, the chronic or excessive production of ROS is recognised as a key mechanism in the progression of inflammatory diseases [13,14].

### 1.2. Lipid Peroxidation and MDA Formation

Lipids, especially polyunsaturated fatty acids containing multiple carbon–carbon double bonds, are the most affected biomolecules in oxidative-stress-induced impairments. At this level, oxidants act by extracting a hydrogen, resulting in the formation of unstable lipid radicals (L-). The subsequent insertion of an oxygen molecule leads to the generation of lipid peroxyl radicals (LOO-), which abstract another hydrogen from a different lipid molecule, continuing the reaction and forming more stable compounds known as lipid hydroperoxides (LOOHs). In this process, called lipid peroxidation, both the lipid hydroperoxides and the lipid peroxyl radicals can undergo cyclization and cleavage processes, resulting in the formation of secondary products [15,16]. MDA is the principal and most extensively studied compound derived from lipid peroxidation, known to possess mutagenic and toxic effects [17]. Additionally, MDA can be enzymatically produced as a side product during the biosynthesis of thromboxane A2 [18].

Once generated, MDA can be metabolised by various enzymes, particularly at the mitochondrial level by aldehyde dehydrogenase, or it can covalently interact with proteins and nucleic acids, leading to the formation of DNA-protein crosslinks and various adducts that damage biomolecules [19,20]. Moreover, a portion of MDA is excreted in the urine [21]. As a result of its interactions, additional modifications occur, leading to the formation of various MDA epitopes that interact with the innate immune system. The effects induced by these MDA epitopes are associated with the expression of pro-inflammatory genes and the activation of several downstream inflammatory signalling pathways, including protein kinase-C, p38-MAPK, ERK1/2 and NF-kB [22].

MDA, as an end product of lipid peroxidation, has been used as a biomarker to measure oxidative stress in various biological samples such as blood, urine and exhaled breath condensate (EBC) in patients affected by a wide range of diseases, including cancer, cardiovascular, pulmonary and neurodegenerative diseases [15,23]. Furthermore, the detection of such end products in inflammatory disorders suggests that lipid peroxidation plays a significant role in this type of disease [22].

**Figure 1:**
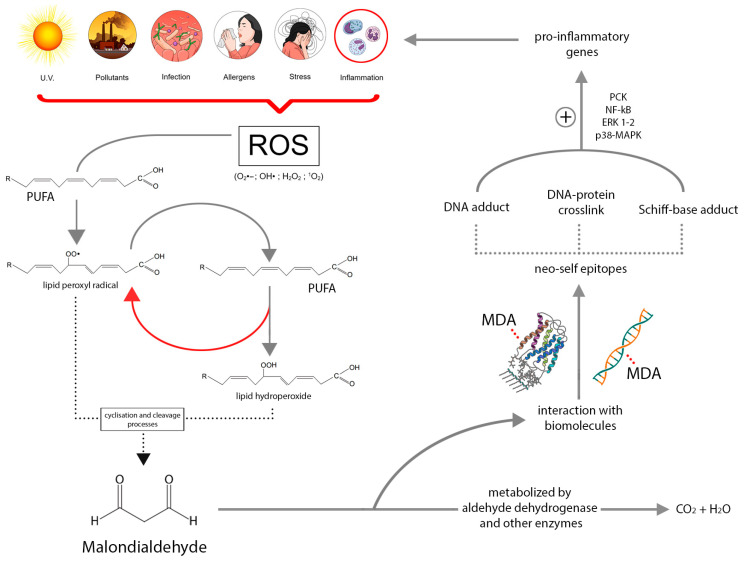
Graphic representation of MDA synthesis and metabolism. The interactions with biomolecules are emphasised to highlight MDA’s role in eliciting inflammation.

Figure 1 describes the main step of MDA formation, metabolism and interactions.

### 1.3. The Role of Oxidative Stress in Allergic Diseases

Oxidative stress and inflammation are prominent features in allergic diseases [24,25,26]. Numerous studies have established a reciprocal amplification between oxidative stress and inflammation in allergic airway diseases [27,28,29,30]. This mutual interaction involves the activation of inflammatory and resident cells, such as eosinophils, neutrophils, monocytes, macrophages, epithelial cells and smooth muscle cells, resulting in significant generation of ROS. This process leads to cellular damage, facilitates the infiltration of additional inflammatory cells into the tissue, triggers the production of pro-inflammatory cytokines and perpetuates a detrimental cycle, ultimately contributing to disease progression [31,32,33].

Oxidative stress also plays a significant role in allergic and inflammatory skin diseases, as highlighted by multiple authors in their reviews [25,34,35]. The skin, being the body’s largest organ, plays a critical role in protecting the body against external threats. However, it is susceptible to oxidative stress caused by reactive species generated in response to environmental and endogenous factors, especially when the skin structure is impaired, such as in atopic dermatitis (AD) [36]. Dysfunctional immune responses to triggers lead to an overproduction of pro-inflammatory cytokines and ROS, resulting in tissue damage and clinical manifestations [37].

Various subsets of lymphocytes are involved in allergic airway and skin diseases, with Th2 being associated with the allergic phenotypes of asthma and rhinitis, as well as the acute phase of AD. Additionally, the Th17 and Th1 subsets contribute to the expression of other asthma phenotypes and the chronicisation of AD lesions. Studies have demonstrated that oxidative stress end products, such as MDA, can influence the polarization of lymphocytes towards Th2 and Th17 subpopulations [38,39], further corroborating the role of oxidative stress in these diseases.

Even in urticaria, the role of oxidative stress is becoming increasingly important. Mast cells and basophils play key roles in the manifestations of urticaria, and their activation leads to the release of histamine, platelet-activating factor and cytokines, which trigger sensory nerve activation, vasodilation, plasma extravasation and the recruitment of cells to urticarial lesions [40]. In vitro investigations have shown that basophils and mast cells can generate ROS when exposed to antigens or anti-FcεRI antibodies. Furthermore, blocking the production of superoxide anions effectively prevents the release of allergic mediators [41]. Additionally, advanced oxidation protein products, which reflect protein impairment due to oxidative stress, were found to be increased in patients with chronic urticaria (CU) [42]. These findings support the concept that oxidative species perform a regulatory role in processes such as mast cell and basophil activation and mediator release.

From the information provided, it is evident that the assessment of the reliable biomarkers of oxidative stress, which can be easily performed and interpreted, is crucial. These biomarkers can play a significant role not only in monitoring disease severity, but also in guiding targeted treatment strategies [43]. Thus, the purpose of our work is to provide an updated overview of the role of MDA as a marker of oxidative stress in allergy-related diseases.

## 2. Results and Discussion

### 2.1. Respiratory

Over the years, numerous authors have extensively investigated the oxidative stress status of patients affected by allergic airway diseases. Various biological samples have been analysed to understand the role of oxidative stress markers. In this context, we specifically focus on MDA as an important factor in the complex pathogenesis of airway diseases, including allergic and non-allergic phenotypes. We aim to evaluate the clinical and laboratory applicability of MDA assessment based on our analysis of the literature. To enhance readability and facilitate data interpretation, the discussion in this section is structured based on the analysed biological samples. We will examine findings from the blood, EBC, bronchoalveolar lavage (BAL) and sputum, with particular emphasis on asthma patients.

#### 2.1.1. Blood

The analysis of MDA levels in blood samples has been extensively employed to investigate the role of oxidative stress in airway diseases. Various studies have examined different methodologies for measuring MDA in this sample, including serum, erythrocyte and lymphocyte MDA levels (S-, E- and L-MDA) [44,45,46,47,48]. However, the accurate measurement of MDA in biological samples can pose challenges due to its instability and susceptibility to degradation. According to some authors [15,48,49], numerous analytical methods exist for determining MDA levels in blood. The most widely recognised method utilises thiobarbituric acid and serves as a basis for detecting MDA and other TBA reactive substances through spectrophotometry. However, pre-analytical factors can impact the results, making precise measurement challenging. The reliable measurement of MDA necessitates special precautions, and comprehensive information regarding analytical and pre-analytical conditions should be provided in scientific reports to ensure result comparability. Other methods such as high-performance liquid chromatography (HPLC), an analytical technique commonly used to separate, identify and quantify components of mixtures, is considered a highly sensitive and specific method for measuring MDA levels in plasma samples. This procedure is accessible to most laboratories equipped with standard HPLC or a spectrofluorometer. Finally, the MDA levels were also determined using the ELISA method, which proved to be sensitive enough to allow for baseline serum determinations [50,51,52].

(a) *Allergic Rhinitis and Asthma*

In the literature, several authors evaluated the oxidative stress status in allergic rhinitis, with most of them including patients with concomitant asthma in their study groups. The largest cohort was analysed by Alsamarai AM et al. [53] based on five surveys involving populations of all ages. They found increased S-MDA values in the affected groups compared to the healthy controls. Additionally, subjects with both asthma and allergic rhinitis exhibited significantly higher S-MDA levels than those with asthma or rhinitis alone, while no differences were observed between the latter two groups. Similarly, Atambay et al. [54] observed elevated E-MDA and L-MDA levels in rhinitis and/or asthmatic patients sensitised and exposed to house dust mites compared to the controls. In contrast, Sagdic et al. [55] reported no differences in the E-MDA levels between the adult patients with rhinitis or asthma and the healthy subjects. Finally, Sadowska-Woda et al. [56] found higher E-MDA levels in children with allergic rhinitis at baseline, and a significant reduction after 2 months of oral desloratadine treatment. 

Based on the evaluation of the aforementioned research, allergic rhinitis appears to contribute to increased oxidative stress, as indicated by elevated levels of S-, E- and L-MDA [53,54,56], although not all studies agree [55]. Oral treatment with antihistamines influences the levels of these oxidative stress markers, leading to a statistically significant decrease in the erythrocyte values [56]. Moreover, the coexistence of both airway diseases appears to exacerbate oxidative stress compared to rhinitis or asthma alone [53].

(b) *Allergic Asthma*

The authors primarily analysed the allergic phenotype of asthma, but none of them evaluated the patients during exacerbations. All the studies found in the literature focused on patients in a relatively stable clinical condition. Two of these studies aimed to evaluate the reduction in oxidative stress biomarkers in adult patients after specific treatments. Specifically, Houseen et al. [57], through a 4-week oral treatment with boswellic acid, curcumin and liquorice in addition to inhalation therapy with inhaled corticosteroids (ICSs) and β2-agonists, achieved a significant reduction in S-MDA levels compared to the placebo group. A similar reduction in S-MDA levels was described by Yalcin et al. [58] in asthmatic patients after biological treatment with omalizumab. Although the control group had higher baseline levels than the pre-treated asthmatic subjects, the post-treatment MDA values with anti-IgE were lower than in all other groups. Among paediatric patients, only Onur et al. [59] conducted a similar study assessing the impact of exercise combined with ICS on S-MDA levels. They found that compared to the baseline, the S-MDA levels were higher in the asthmatic patients than in the controls. However, no statistically significant changes were observed between the affected group treated only with inhalation therapy and those who received a combination of therapy and exercise. Fabian et al. [60] also found increased basal levels of S-MDA in asthmatic children, which was negatively correlated with forced expiratory volume in the first second (FEV1) and was positively correlated with the Fraction of Exhaled Nitric Oxide (FENO). On the other hand, Petlevski et al. [61] did not find any differences in the paediatric cohort receiving inhalation therapy with ICS and/or β2 agonists. Two articles [62,63] examined controlled and uncontrolled asthma patients based on their Asthma Control Test (ACT) values, symptom frequency, use of bronchodilators and FEV1 values. Both studies reached the same conclusion: the S-MDA levels were higher in the uncontrolled/poorly controlled groups. Furthermore, one of the studies [63] found a negative correlation between the S-MDA and ACT values and, as reported by Fabian et al. [60], with the FEV1 values.

It can be concluded that the allergic asthma patients, particularly those with poorly controlled disease [62,63], have increased levels of S-MDA compared to the healthy controls [58,59,60]. However, in paediatric subjects, not all studies agree [61]. Additionally, some studies have shown relationships between MDA concentrations and lung function parameters, particularly a negative association with FEV1 values and ACT values, and a positive correlation with FENO concentrations [60,63], supporting the concept of clinical and laboratory utility for these oxidative stress markers. Finally, exercise does not seem to reduce the MDA levels when used alongside pharmaceutical treatments, while inhalation therapies [59,62], biological therapies [58] and compounds from natural extracts [57] seem to have beneficial effects on reducing oxidative stress in the airways.

(c) *Non-Allergic Asthma and mixed cohort*

Some authors included a mixed cohort of allergic and non-allergic asthmatic patients in their analysis. Anes et al. [64] found higher levels of S-MDA in stable asthmatic adults receiving treatment with ICS and β2-agonists compared to the healthy controls. The same increase was found by Ercan et al. in an untreated paediatric cohort [65], and by Ammar et al. [66] in asthmatic adults with controlled/uncontrolled disease according to ACT scores. Furthermore, the studies indicated that the higher the S-MDA values, the more severe the asthma and the worse its control [65,66].

“Intrinsic” asthma was evaluated during both the stable and exacerbation periods of the disease. Celiak et al. [67] found increased E-MDA levels and reduced erythrocyte glutathione (E-GSH) levels in non-allergic stable asthmatic adult patients compared to the healthy controls. Meanwhile, Tug et al. [68] examined a group of adult patients receiving different therapies for four weeks after an acute asthma attack. At baseline, all patients had higher levels of S-MDA than the controls, and after the treatment, the main significant reduction in these levels was found in Group I, which received inhaler β2 agonists + inhaler budesonide + oral leukotriene receptor antagonists. Overall, no differences were found between the affected groups after the treatments. Cheng et al. [69] studied the serum levels of MDA, IL-25 and thymic stromal lymphopoietin in children during asthma exacerbations. Their results show that the abundance of particulate matter 2.5 and dust mite antigens inducing asthma exacerbations resulted in higher serum levels of the studied markers, even after the resolution of the acute attacks, compared to the “standard” concentrations of these exacerbation triggers.

Based on the considered cohorts of non-allergic and mixed asthmatic patients, there is a clear consensus among all authors: the MDA levels assessed in the blood samples were found to be elevated in the affected patients [64,65,66,67,68,69]. Furthermore, these levels were associated with increased asthma severity and poorer disease control [65,66]. However, in contrast to other authors, a recent article found no such increase in a mixed cohort of paediatric patients with well-controlled asthma [70]. Ultimately, different treatment regimens do not appear to significantly differ in their effectiveness in reducing MDA levels, although combination therapy with inhaled corticosteroids, β2 agonists and anti-leukotrienes seems to have a more pronounced impact.

(d) *Phenotype not specified*

Unfortunately, most of the studies in the literature did not describe the phenotype and clinical period of the asthmatic patients examined, making the results less usable for comparative purposes. Ozaras et al. [71] found higher levels of S-MDA in asthmatic adults compared with the controls, and after one month of treatment with ICS and inhaled β2 agonists compared to baseline. The same increase was reported by Narula et al. [72] in asthmatic children in a stable clinical period compared to healthy controls. Higher levels of S-MDA were also observed during exacerbations in adult patients compared to the controls [73], and in obese asthmatic patients compared to non-obese patients [74]. Furthermore, the latter study showed a positive correlation between the MDA levels and the frequency of severe acute exacerbations in obese patients. Also, in children with exacerbations, the S-MDA levels were increased compared to the healthy controls [75,76]. In one of these studies [75], higher MDA levels were correlated with more severe asthma exacerbations. A comparison of the S-MDA levels between exacerbations and periods of stability was performed by some authors, finding higher levels during acute attacks in both adult [77,78] and paediatric [79] cohorts. Finally, even in the studies lacking definitions of the phenotype and clinical period of asthma patients, the S-MDA levels were higher in the patients than in the controls [80,81], although no correlation with lung function was found in children [82]. Moreover, one of these studies [80] showed increased MDA levels even in salivary samples.

Although not optimally detailed from the point of view of phenotyping and the description of the clinical period, the studies described here strongly emphasise the concept of increased serum MDA in asthma patients, whether adult [71,73,74,77,78,80,81], paediatric [72,75,76,79,82], during exacerbation or clinically stable. Certain factors, such as obesity and severe exacerbations themselves, were correlated with higher levels of MDA [74,75]. However, in this cluster of studies, there was no correlation with lung function. Finally, treatment with ICS led to a reduction in the levels of S-MDA [76].

The characteristics of all the blood samples from the studies discussed above are reported in Table 1.

#### 2.1.2. Bronchoalveolar Lavage (BAL)

BAL is recognised as the most reliable method for sampling the lining fluid of the lower respiratory tract. It allows for the assessment of immune cells, microorganisms, inflammatory cytokines and mediators of oxidative stress in the airways and alveolar spaces of patients with various pulmonary diseases. However, the invasive nature of this procedure and the requirement for sedation limit its applicability [83,84]. 

Some authors have used BAL to assess the oxidative stress status in asthma patients, particularly by analysing the MDA levels alone or in conjunction with other compounds. However, these studies were conducted during periods of relative clinical stability and not during exacerbations. In one study, Brown et al. [85] evaluated the oxidative stress status in mild-to-moderate and severe asthmatic children compared to non-asthmatic atopic adults. The BAL analysis revealed increased concentrations of MDA, IL-13 and 8-isoprostane in asthmatic patients compared to the control subjects. Furthermore, the MDA concentrations increased with the asthma severity. Another study [86] found increased MDA concentrations in atopic children with severe asthma, although the control group consisted of both healthy adults and children. In contrast, Schock et al. [87] did not find significantly different MDA levels or other antioxidants (ascorbate, urate and α-tocopherol) in the BAL between the affected children and the age-matched control group. However, the lack of homogeneity in the control group, which included both atopic and non-atopic children, makes comparisons difficult. Ozaras et al. [71] conducted a study on asthmatic adults, evaluating the MDA levels in the BAL and serum (previously described in the Section 2.1.1) before and after one month of inhalation treatment with ICS and beta2-agonists. The results show a negative correlation between the MDA and FEV1 levels before the treatment and a significant reduction in the MDA levels after one month of treatment in the patients compared to the baseline values. However, the authors did not specify the asthma phenotype. 

Although some studies on atopic children indicate increased MDA levels in BAL [85,86], the lack of homogeneity in the control population prevents clear comparisons. The only study [87] that considered the demographic characteristics of the controls found no significant differences in the MDA levels in the BAL of the asthmatic and non-asthmatic patients. However, this study did not account for the presence or absence of atopy in the controls. Furthermore, inhalation therapy in asthmatic adults seems to have a beneficial effect on the local pulmonary oxidative state of patients [71]. It is important to note that the aforementioned studies did not consider the exacerbations of the disease. In conclusion, the limited number of studies and the lack of cohort homogeneity do not allow for a conclusive analysis or clear recommendations. The major limitation lies in the complexity and invasiveness of performing BAL, making it challenging to apply in clinical practice for monitoring the oxidative and inflammatory status of patients with respiratory allergic diseases.

#### 2.1.3. Exhaled Breath Condensate (EBC)

To address the limitations associated with BAL sampling, new minimally invasive procedures have been developed over time. One such procedure is the evaluation of the EBC, which is obtained by cooling exhaled air. An EBC analysis is considered a good, non-invasive and easy method for monitoring the inflammation and oxidative stress in the lower airways [88]. It has the potential to be useful in monitoring therapeutic response and identifying specific biomarkers for lung diseases [89]. Numerous authors have used EBC to analyse the composition of the airway lining fluid in different pathological conditions, including the evaluation of oxidative stress markers such as MDA [90].

Several studies have evaluated the MDA levels in EBC from both paediatric and adult asthmatic patients. However, not all articles specified the asthma phenotype or severity, and some studies included mixed cohorts with other lung diseases or concomitant allergic rhinitis. Aksu et al. [91] evaluated adults with asthma alone (including both atopic and non-atopic subjects), rhinitis alone and both airway diseases compared to a healthy control group. The authors found no significant differences in the MDA levels between the patient groups or compared to the controls. On the other hand, Celik et al. [92] studied three groups of affected atopic children: asthmatics with allergic rhinitis, asthmatics without allergic rhinitis and non-asthmatics with allergic rhinitis. They found higher MDA levels in the oral and nasal EBC in all patients compared to the controls. It is worth noting that the oral MDA levels were higher in the patients with allergic asthma alone than in the patients with both allergic airway diseases. Dut et al. [93] assessed allergic asthma in children during stable periods of the disease and found increased MDA levels compared to the controls. Lärstad et al. [94] studied atopic subjects with and without allergic asthma but did not find significant differences, although they did not specify the clinical period. Corradi et al. [95] included both asthma phenotypes in their study on paediatric patients during disease exacerbations. The authors reported increased MDA levels in the affected cohorts, which is consistent with the findings of Dut et al. [93] and Celik et al. [92]. Additionally, they showed that five days of oral prednisone therapy resulted in the MDA values no longer differing from those of the control subjects. The GSH levels remained low in the asthmatic patients compared to the healthy controls both before and after treatment. Romieu et al. [96] used the MDA levels in the EBC of allergic and non-allergic asthmatic children as markers of exposure to traffic pollution. The results show higher MDA levels in relation to an increased exposure to traffic-related pollutants, and these levels were inversely associated with the FEV1 and forced vital capacity values, while they were directly correlated with the IL-8 levels in the nasal lavage. Finally, Bartoli et al. [97] found increased MDA levels in the EBC of asthmatic adults compared to the controls. The corticosteroid-treated asthmatic patients had lower MDA levels than the untreated patients. It is important to note that this article did not define the phenotype of asthmatic patients and included subjects with bronchiectasis, chronic obstructive pulmonary disease and idiopathic pulmonary fibrosis. Except for the latter, increased levels of MDA were found in all patients, and chronic obstructive pulmonary disease patients had the highest levels.

In summary, increased MDA levels and decreased GSH levels were found in the EBC of atopic children with asthma and/or rhinitis during both the remission [92,93] and exacerbation [95] periods. However, the presence of both allergic conditions does not appear to further increase oxidative stress as measured via MDA analysis in the EBC [92]. Furthermore, external factors such as traffic-related pollution can negatively affect the oxidative stress states and pulmonary function of patients [96]. Oral corticosteroid therapy during exacerbations seems to be helpful in reducing the MDA levels but not the glutathione levels in the short term [95]. On the contrary, in adult cohorts, the presence of allergic airway diseases does not appear to increase oxidative stress in terms of detectable MDA levels in EBC [91,94]. However, one study conducted on stable asthmatic adults found increased MDA levels, although the authors did not specify the phenotype [97].

The key characteristics of the studies evaluating the BAL and EBC samples are shown in Table 2.

### 2.2. Cutaneous

The skin hosts a wide range of enzymatic and non-enzymatic compounds that act as antioxidants or oxidant-degrading molecules. The involvement of oxidative stress in the pathogenesis of allergic skin disorders has been speculated for decades. However, few clinical studies have evaluated oxidative stress in allergic skin diseases due to difficulties in measuring the levels of oxidative stress markers. The most reliable and simplest biological samples to take were blood, urine and EBC. In this section, the results for CU/AU and AD will be reviewed. Our discussion focuses on the role of MDA as a possible tool in the clinical and laboratory practice of allergic skin diseases, as oxidative stress is recognised as one of the main contributors to this type of disorder [25].

#### 2.2.1. Urticaria

Urticaria is a common skin manifestation with several aetiologies, such as the allergy to food and drugs, autoimmunity phenomena, acute and chronic infections and many others. Based on the duration of symptoms (more or less than 6 weeks), urticaria is classified into acute and chronic. CU can be classified into an inducible form and a spontaneous form (chronic idiopathic urticaria/chronic spontaneous urticaria CIU/CSU) [98]. The latter was defined as an “auto-allergic condition” based on the presence of the so-called highly cytokinergic IgE, and an “auto-immune condition”, where it is mediated by autoreactive IgG [99]. The role of oxidative stress in urticaria has been extensively studied [35]. In fact, different types of urticaria have been investigated by assessing the oxidative stress state in affected patients.

One of the main aspects investigated by researchers is the measurement of MDA levels in blood or skin samples and their association with CIU/CSU. A study reported increased MDA levels in the blood samples of CU patients compared with those of the healthy controls, whereas another serum marker of lipid peroxidation, 4-hydroxy-2-nonenal (4-HNE), did not differ between the two groups [100]. The same study also involved patients with AD with different results (discussed in the dedicated section). On the contrary, other studies reported opposite results. Kasperka-Zajac et al. [101] reported no statistically significant differences in the levels of S-MDA and E-MDA, as well as in the activity of specific antioxidant compounds, between healthy subjects and patients with CIU, regardless of their positivity or negativity in the autologous serum skin test (ASST). Similarly, the activities of copper-zinc superoxide dismutase (CuZn/SOD) and manganese superoxide dismutase (Mn/SOD) in plasma and CuZn/SOD, GSH/Px and CAT in erythrocytes did not differ between the study groups. Similar results were obtained by Sagdic et al. [102], who evaluated E-MDA levels and GSH/Px activities among CIU patients and healthy controls. Conversely, a statistically significant decrease in CuZn/SOD erythrocyte activities was reported in CIU patients. In the most recent report by Rajappa et al. [103], the platelet MDA levels were significantly elevated in CSU patients, while the platelet SOD and GSH/Px levels were significantly reduced compared to the controls. Furthermore, these changes were related to a worse urticaria severity score. Finally, only one study assessed the MDA levels and SOD and GSH activities in the tissue samples from a punch biopsy of wheals and healthy skin of patients with CIU [104]. Although no significant differences were observed between the unaffected samples of the CIU and healthy control samples, the affected skin samples showed significantly elevated levels of MDA, as well as increased activities of SOD and GSH, in comparison to both the non-affected patient samples and the control samples. In addition, an upregulation of Mn/SOD was reported in affected skin tissue as a compensatory mechanism of mitochondrial oxidative stress.

The evaluation of some lipid peroxidation parameters and the status of antioxidant enzymes were also performed in AU patients, mainly during adverse drug reactions.

During the acute phase of cutaneous adverse drug reactions, Verma et al. [105] observed higher levels of MDA and lower levels of GSH in the serum of patients compared to the control group, which did not experience adverse reactions while taking the offending drugs. Another study on AU showed significantly elevated serum MDA levels in the patients compared with the controls [106]. Lastly, the study performed by Kasperka-Zajac et al. [107] involved patients with urticaria, with or without angioedema, caused by NSAIDs, and control subjects to determine some of the indirect parameters of ROS activity in serum and erythrocytes. The results show no statistically significant differences between the two groups regarding the MDA levels, as well as some antioxidant compounds, in the serum and erythrocyte.

In the considered studies, a drug allergy is reported to be the main causal agent in AU patients [105,106,107]. The pooled results raise the possibility that the serum and erythrocyte MDA levels, as well as those in the chronic form, increase during the disease [100,103,104,105,106], although not all the studies agree [101,102,107]. Moreover, in CIU/CSU patients, the S- MDA and E-MDA levels do not seem to be related to disease activity or severity, as only one study found a clear correlation [103]. Oxidative stress certainly plays an important role in urticaria, but the specific function of MDA remains uncertain. Therefore, lipid peroxidation needs to be further investigated in urticaria patients, in terms of analysing the MDA levels in different biological samples and standardizing procedures to obtain a complete utility profile of this marker in the disease under consideration.

The key findings of the considered studies are summarised below in Table 3.

#### 2.2.2. Atopic Dermatitis

Like other allergic disorders, recent clinical and basic approaches have shown that AD is a heterogeneous condition and can be classified into different types. In particular, based on the presence or absence of IgE antibodies to environmental allergens, generally mite-derived allergens, extrinsic/allergic and intrinsic/non-allergic AD are identified. Extrinsic AD is often accompanied by respiratory and food allergies, and since many patients with intrinsic AD exhibit hypersensitivity to metals rather than to protein antigens, the term “non-allergic” is also problematic for this type of AD [108]. However, the two main features of AD are inflammation and redox imbalance, which mutually interact and lead to its clinical manifestations.

Several authors have assessed the markers of oxidative stress and enzyme activities in AD patients, and according to a recent review, MDA is the oxidative stress marker that is more frequently measured in various types of samples in affected patients [37]. In one study, S-MDA in all-age AD patients were found to be elevated compared to the healthy controls together with reduced vitamin A and E levels [109]. On the contrary, another study, which was previously mentioned in the Section 2.2.1 [100], reported no statistically significant differences between all-age patients and healthy controls; the same result was reported for another serum marker of lipid peroxidation, 4-HNE. In terms of children’s AD population, a study reported high, but not statistically significant, S-MDA levels, while the total antioxidant capacity was significantly reduced [110]. Other works found no differences in the S-MDA levels [111,112] and U-MDA [112] compared to the healthy controls. However, one of these studies showed a positive correlation between the malondialdehyde/melatonin ratios and the severity of disease [111]. Another study on urine samples was performed with an adult population, showing no differences in the MDA levels between the patients and controls; however, the MDA levels were significantly correlated with the disease severity [113]. Finally, MDA was evaluated in the EBC [114] of AD children. Unexpectedly, the MDA levels were higher, although not statistically significant, in the control group.

Although in some studies, the MDA levels were increased in the affected patients [109,110] and were associated with a more severe disease [111,113], the role of this oxidative stress marker in AD patients remains controversial. Indeed, the serum [100,111,112], urinary [112,113] and EBC [114] assessments have not shown significant increases in the MDA levels in the affected patients. This could be related to the presence of a few studies conducted on the same biological sample but also, in one specific case [112], to the lack of a control group. In view of the potential applications of MDA levels in AD patients as a measure of the diseases’ severity and for monitoring during certain therapies, further research is required to broaden the case series that are already available and provide solid and useful recommendations.

Table 4 summarises the main findings from the studies considered.

## 3. Materials and Methods

We conducted a PubMed search using the following keywords: “malondialdehyde”, “asthma”, “rhinitis”, “urticaria”, “atopic dermatitis”, “allergic contact dermatitis”, “hymenoptera venom allergy”, “anaphylaxis” and “food allergy”. Our analysis included all research articles in English and conducted on humans, without a time limitation, that explored the involvement of MDA, alone or in combination with other oxidative stress markers or enzymes, in allergic diseases.

## 4. Conclusions

The involvement of oxidative stress in allergic inflammatory airways and skin diseases is well established, and several of the biomarkers were studied with the aim of increasing the existing knowledge of the mechanisms involved in disease development and progression. This review article focuses on the current information on MDA as a biomarker of oxidative stress in allergic pathologies. These conditions are characterised by a high incidence, mainly in developed countries, and are associated with relevant morbidity and/or mortality, causing a significant alteration in the quality of life.

The data reported in this review highlight the role of MDA in inflammatory airway diseases. Blood is the most used sample, and the majority of studies show elevated MDA levels in both allergic and non-allergic asthma phenotypes. Moreover, the oxidative stress status in asthmatic patients seems to worsen in the presence of other allergic airway diseases, and in relation to disease severity. The most common asthma treatments positively influence both the inflammatory status and oxidative stress status. Thus, the determination of the MDA levels can be a valuable biomarker for monitoring the status of allergic airway diseases. However, the reported studies show a big inhomogeneity and several limitations caused by the lack of phenotyping and description of the clinical period of the patients examined. Furthermore, the behaviour of MDA in the presented studies can sometimes be related to a series of physio/pathological conditions, such as physiological factors (e.g., age), environmental conditions (e.g., pollution), lifestyle habits, treatment regimens (on medication or not) and the presence of co-morbidity (e.g., obesity). Finally, other samples, such as the BAL and EBC, are poorly studied, and the results are not univocal. 

In cutaneous allergic diseases, the role of MDA is controversial due to the discordance that is present in the reported studies. Clearer positions can be taken regarding various forms of urticaria, as most studies based on serum assessments have shown an increase in the MDA levels in the affected patients compared with the controls. However, in the case of atopic AD, the evidence is not unequivocal in supporting such an increase. Several limitations may arise in studies focused on skin assessment, primarily due to the smallness of the studies and the heterogeneity of the samples (blood, urine and skin).

In conclusion, MDA appears to be a valid biomarker of oxidative stress in allergic inflammatory airway diseases. Nevertheless, based on the available data in the literature, a clear conclusion regarding its applicability in allergic skin diseases cannot be formulated. Further studies on larger cohorts of patients with better defined variables in terms of diagnostic definitions, standardization of the methods and homogenization of the clinical characteristics can give more significant results. This can help researchers to better understand the mechanisms that are involved in the development and progression of allergic diseases, to select patients with the worst clinical conditions and guide them to targeted therapies.

## Figures and Tables

**Table 1:** Features of studies assessing MDA in blood from individuals with asthma and/or rhinitis.

BLOOD							
*Diseases*	*Authors*	*Population*	*Cohort (Controls)*	*Baseline Treatment*	*Outcomes*	*Findings*	*Methods of MDA Detection*
***Allergic Rhinitis and/or Asthma***							
	Alsamarai AM et al. [53]	All	16,636 (14,414)	NS	Association between AR and asthma	↑ S-MDA levels (*p* < 0.0001 in all patients vs. CS; *p* < 0.0001 in A + AR vs. AR or A); no differences in MDA levels between A and AR	Spectrophotometry
	Atambay et al. [54]	All	90 (30)	Not on medication	Assessment of the relation between cellular enzymatic antioxidant capacity and the degree of membrane lipid peroxidation	↑ E-MDA levels (*p* < 0.05 in DM+/SPT+ vs. DM-/SPT+ and CS); ↑ L-MDA levels (*p* < 0.05 in DM+/SPT+ and DM-/SPT+ vs. CS)	Spectrophotometry
	Sagdic et al. [55]	Adults	94 (36)	On medication with ICS	Role of oxidative stress in etiopathogenesis of allergic diseases	No differences in E-MDA levels	Spectrophotometry
	Sadowska-Woda et al. [56]	Children	50 (11)	Not on medication for at least 1 month	Evaluation of oxidative stress parameters before and after 2-month treatment with systemic desloratadine	Before treatment: ↑ E-MDA levels (*p* < 0.001 in UnTxAR vs. CS) After treatment: ↓ E-MDA levels (*p* < 0.001 in TxAR vs. UnTxAR)	Spectrophotometry
***Allergic Asthma***							
*Stable*	Houseen et al. [57]	Adults	63 (24)	On medication with ICS and B2 agonists	Efficacy of 4-week treatment with boswellic acid, curcumin and liquorice in asthma management	↓ S-MDA levels (*p* < 0.001 in TxA vs. PlA)	Spectrophotometry
	Yalcin et al. [58]	Adults	42 (14)	On medication with biological drug	Evaluation of the effect of omalizumab treatment on oxidative stress status	Before treatment: ↑ S-MDA levels (*p* < 0.0001 in CS vs. A); after treatment: ↓ S-MDA levels (*p* < 0.0001 in TxA vs. UnTxA and CS)	Spectrophotometry
	Onur et al. [59]	Children	43 (13)	Not on medication for at least 1 month	Beneficial effect of exercise in addition to inhaled fluticasone treatment for 2 months on antioxidant status	Before treatment: ↑ S-MDA levels (*p* = 0.0001 in A vs. CS); after treatment: ↓ S-MDA levels (*p* < 0.001 in PTxA and P + ExTxA vs. pre-TxA values); no differences in S-MDA levels (*p* > 0.05 in PTxA vs. P + ExTxA vs. post-TxA)	Spectrophotometry
	Fabian et al. [60]	Children	56 (21)	Not on medication for at least 1 month	Assessment of the relationship between antioxidant enzyme activities, airway inflammation and systemic oxidative stress	↑ S-MDA and ↑ IL-6 levels (*p* < 0.001 in A vs. CS); Positive correlation between S-MDA levels, IL-6 levels and FENO values (*p* = 0.002; *p* = 0.001); negative correlation between S-MDA and FEV1 values (*p* = 0.005)	HPLC
	Petlevski et al. [61]	Children	81 (37)	On medication with ICS or ICS plus long-acting b2-agonists	Comparison of oxidative stress and lipid peroxidation markers	No differences in S-MDA and GSH levels	Spectrophotometry
*Stable/poorly controlled based on symptoms, bronchodilator use* *and FEV1 levels*	Al Obaidi et al. [62]	Adults	153	Not on medication	Evaluation of oxidative stress parameters to assess the effectiveness of ICS and salbutamol therapy for 4 weeks	↑ S-MDA (95% CI 6.98–7.88 in PoCA vs. 4.03–4.23 in StA)	Spectrophotometry
*Controlled/uncontrolled based on ACT*	Karadogan et al. [63]	Adults	240 (120)	Not/on medication with ICS	Evaluation of oxidant/antioxidant status and the relation with the level of asthma control	↑ S-MDA, ↓ GSH levels (*p* < 0.001 in A vs. CS); ↑ S-MDA levels (*p* < 0.01 in UnCA vs. CA; *p* < 0.05 in UnCA vs. PCA); negative correlation between S-MDA levels, ACT scores and FEV1 values (*p* = 0.002; *p* = 0.017)	Spectrophotometry
***Allergic/Non-Allergic Asthma***							
*Stable*	Anes et al. [64]	Adults	329 (178)	On medication with ICS and/or inhaled beta-2 agonist	Assessment of oxidant/antioxidant status and its relation to disease progression and decline in lung function	↑ S-MDA and ↓ GSH levels (*p* < 0.001 in A vs. CS)	Spectrophotometry
	Ercan et al. [65]	Children	567 (255)	Not on medication	Definition of the factors affecting oxidative stress levels	↑ S-MDA and ↓ GSH levels (*p* < 0.001 in MA vs. CS and in MSA vs. MA); asthma severity influences MDA and GSH levels (*p* < 0.001)	HPLC
*Controlled/uncontrolled based on ACT*	Ammar et al. [66]	Adults	108 (48)	On medication with ICS and oral or inhaled beta2-agonist	Evaluation of oxidant/antioxidant status and its relation to the level of asthma control	↑ S-MDA, ↑ AOPP, ↓ GSH levels (*p* < 0.001 in A vs. CS); ↑ S-MDA (*p* < 0.001 in UnCA vs. CA)	Spectrophotometry
***Non-Allergic Asthma***							
*Stable*	Ceylan et al. [67]	Adults	60 (30)	On medication with ICS and short-acting b2 agonist	Correlation between L-arginine–NO pathway, asthma and oxidative stress	↑ E-MDA levels (*p* < 0.001 in A vs. CS); ↓ E-GSH levels (*p* < 0.01 in A vs. CS)	Spectrophotometry
	Tug et al. [68]	Adults	41 (10)	Not on medication	Effect of different treatment regimens on oxidative stress markers and inflammation	Before treatment: ↑ S-MDA levels (*p* < 0.005 in all patients vs. CS); after treatment: ↓ S-MDA levels (*p* < 0.005; *p* < 0.01; *p* < 0.05 in Groups I, II and III, respectively)	HPLC
*Exacerbations*	Cheng et al. [69]	Children	96 (24)	NS	Assessment of the influence of PM2.5 and Der p1 on the treatment of asthma attacks	↑ S-MDA, IL-25 and TSLP levels (*p* < 0.001 in ExAE vs. CAE after the treatment)	ELISA
***Phenotype Not Specified***							
*Stable*	Ozaras et al. [71]	Adults	38 (24)	Not on medication for at least 6 months	Evaluation of respiratory function and lipid peroxidation markers before and after 1 month of steroid and beta2-agonist inhaler therapy	Before treatment: ↑ S-MDA levels (*p* < 0.001 in A vs. CS) After treatment: ↓ S-MDA levels (*p* < 0.001 in TxA vs. baseline); ↑ S-MDA levels (*p* < 0.0001 TxA vs. CS)	HPLC
	Narula et al. [72]	Children	311 (156)	NS	Identification of the extent of lipid peroxidation with asthmatic severity	↑ S-MDA levels (*p* < 0.01 in A vs. CS)	Data not available
*Exacerbations*	Jacobson et al. [73]	Adults	30 (15)	On medication with ICS and/or other controller drugs for at least 24 h	Evaluation of plasmatic oxidative stress in acute severe asthma patients	↑ S-MDA levels (*p* < 0.05 in AE vs. CS)	Spectrophotometry
	To et al. [74]	Adults	49	On medication	Evaluation of the potential effect of systemic oxidative stress on acute exacerbations in obese asthmatic patients	↑ S-MDA levels (*p* < 0.05 in OA vs. NOA); positive correlation between S-MDA levels and frequency of severe acute exacerbations in OA	Spectrophotometry
	Al-Abdulla et al. [75]	Children	219 (121)	Not on medication	Assessment of oxidative stress state during asthma exacerbations and its possible correlation with attack severity	↑ S-MDA levels (*p* < 0.001 in AE vs. CS); correlation between S-MDA levels and severe asthma exacerbations (*p* < 0.001)	Spectrophotometry
	Owayed et al. [76]	Children	35 (18)	Not on medication	Evaluation of the effect of salbutamol and NO on the NADPH oxidase system of PBL	↑ S-MDA levels (*p* < 0.05 in AE vs. CS); ↓ L-MDA levels (*p* < 0.01 in STxPBL vs. UnTxPBL)	Spectrophotometry
*Stable/exacerbations*	Fatani et al. [77]	Adults	90 (30)	NS	Assessment of the oxidant/antioxidant balance between asthmatic patients (during acute attacks and stable period) and control subjects	↑ S-MDA levels (*p* < 0.001 in AE vs. CS and in AE vs. StA; *p* < 0.01 in male StA vs. male CS; *p* < 0.001 in female StA vs. female CS)	Spectrophotometry
	Gumral et al. [78]	Adults	32	NS	Assessment of the oxidant–antioxidant status during the exacerbation and the stable period in patients with asthma or COPD	↑ S-MDA levels (*p* < 0.01 in AE vs. StA)	Spectrophotometry
	Leem et al. [79]	Children	16	Not/on medication with ICS	Association between pulmonary inflammation and environmental oxidants and tobacco smoke	↑ S-MDA levels (*p* = 0.006 in AE vs. StA)	HPLC
*Not specified*	Abboud et al. [80]	All	205 (102)	NS	Investigation of the pathogenicity of a group of oxidative stress by-products in asthmatic disease	↑ S-MDA levels (*p* < 0.05 in A vs. CS) In saliva: ↑ MDA levels (*p* < 0.05 in A vs. CS)	NS
	Ruprai et al. [81]	Adults	80 (40)	On medication	Assessment of oxidants/antioxidants and their correlation with pulmonary function	↑ S-MDA levels (*p* < 0.001 in A vs. CS); no correlation between S-MDA levels and pulmonary function test	Spectrophotometry
	Shabestari et al. [82]	Children	75 (25)	NS	Comparison of the oxidative stress markers in asthmatic + CAP and CAP only in children	↑ S-MDA levels (*p* < 0.001 in A + CAP vs. CAP and CS)	Spectrophotometry

↑ = increased; ↓ = decreased; A = asthmatic patients; AR = allergic rhinitis patients; CS = control subjects; UnTxAR = untreated allergic rhinitis patients; TxAR = treated allergic rhinitis patients; UnTxA = untreated asthmatic patients; TxA = treated asthmatic patients; E-MDA = erythrocyte MDA; S-MDA = serum MDA; L-MDA = lymphocyte MDA; DM+/SPT+ = dust mite+/skin prick test+ patients; DM-/SPT+ = dust mite-/skin prick test+ patients; PTxA = pharmacologically treated asthmatic patients; P + ExTxA = pharmacologically treated and exercise-treated asthmatic patients; UnCA = uncontrolled asthmatic patients; PCA = partially controlled asthmatic patients; CA = controlled asthmatic patients; ExAE = exposed asthma exacerbation patients; CAE = controlled asthma exacerbation patients; PoCA = poorly controlled asthmatic patients; StA = stable asthmatic patients; PlA = placebo asthmatic patients; MA = mild asthmatic patients; MSA = moderate–severe asthmatic patients; AE = asthma exacerbation patients; STxPBL = salbutamol-treated peripherical blood lymphocyte; UnTxPBL = untreated peripherical blood lymphocyte; OA = obese asthmatic patients; NOA = non-obese asthmatic patients; CAP = community-acquired pneumonia patients; HPLC = high-performance liquid chromatography; NS = not specified.

**Table 2:** Features of studies assessing MDA in the BAL and EBC from individuals with asthma and/or rhinitis.

BAL							
*Diseases*	*Authors*	*Population*	*Cohort (Controls)*	*Baseline Treatment*	*Outcomes*	*Findings*	*Methods of MDA Detection*
***Allergic Asthma***							
*Stable*	Brown et al. [85]	Children	80 (12 atopic adults)	On medication for at least 6 weeks with ICS or systemic corticosteroids and/or other additional controller drugs	Evaluation of lipid peroxidation markers, pro-inflammatory cytokine and airflow limitation	↑ MDA (*p* < 0.01 in SA vs. CS; *p* < 0.05 in SA vs. MMA), IL-13 (*p* < 0.01 in SA vs. CS; *p* < 0.05 in MMA vs. CS) and 8-isoprostane levels (*p* < 0.001 in SA vs. CS; *p* < 0.001 in MMA vs. CS)	Spectrophotometry
	Fitzpatrick et al. [86]	Children	106 (6 children and 35 adults)	On medication for at least 8 weeks with ICS or systemic corticosteroids and/or other additional controller drugs	Assessment of airway oxidative stress status	↑ MDA, 8-isoprostanes and H_2_O_2_ levels (*p* < 0.05 in A vs. CS)	Spectrophotometry
	Schock et al. [87]	Children	202 (83 non-atopic and 41 atopic children)	On medication with ICS or systemic corticosteroids and/or other additional controller drugs	Assessment of the antioxidants and oxidated protein concentration	No differences in MDA levels, antioxidants, and oxidated protein concentration	HPLC
***Phenotype Not Specified***							
*Stable*	Ozaras et al. [71]	Adults	14	Not on medication for at least 6 months	Correlation with respiratory function and lipid peroxidation markers before and after 1 month of ICS and beta2-agonist inhalers therapy	Before treatment: negative correlation between MDA and FEV1 levels (*p* < 0.05 in A); after treatment: ↓ MDA levels (*p* < 0.001 in TxA vs. baseline)	HPLC
**EBC**							
***Diseases***	***Authors***	***Population***	***Cohort (Controls)***	***Baseline Treatment***	***Outcomes***	***Findings***	***Methods of MDA Detection***
***Asthma and/or Rhinitis***							
*A (atopic and non-atopic—stable) and/or Rhinitis*	Aksu et al. [91]	Adults	94 (13)	Not on medication	Evaluation of lower airway inflammation status and the influence of atopy and eosinophilia on MDA levels	No differences in MDA levels	Spectrophotometry
*A (atopic—stable) and/or AR*	Celik et al. [92]	Children	219 (74)	Not on medication for at least 1 week with antihistamines, 4 weeks with systemic corticosteroids and 3 days with antileukotrienes	Determination of oxidative stress levels in nasal and oral EBC	↑ MDA levels and ↓ GSH levels in both oral and nasal EBC (*p* < 0.01 in MMA + AR, AR, MMA vs. CS); ↑ MDA levels (*p* < 0.01 in A vs. MMA + AR)	HPLC
***Allergic Asthma***							
*Stable*	Dut et al. [93]	Children	331 (191)	Not on medication	Characterization of the oxidant/antioxidant imbalance	↑ MDA levels (*p* < 0.01 in MoA vs. CS); ↓ GSH levels (*p* < 0.001 in MoA vs. CS)	HPLC
*Not specified*	Lärstad et al. [94]	Adults	44 (15 atopic adults)	NS	Determination of MDA levels	No differences in MDA levels	HPLC
***Allergic/Non-Allergic Asthma***							
*Exacerbations*	Corradi et al. [95]	Children	22 (10)	On medication for at least 2 months with ICS (*n* = 10); not on medication (*n* = 2)	Determination of oxidant/antioxidant levels at exacerbation and after 5 days of oral prednisone therapy	Before treatment (AE vs. CS): ↑ MDA levels (*p* = 0.002); ↓ GSH levels (*p* < 0.001); after treatment: ↓ MDA levels (*p* = 0.001 in TxAE vs. AE); ↑ GSH (*p* = 0.04 in TxAE vs. AE); ↓ GSH (*p* < 0.001 in TxAE vs. CS)	HPLC
*Not specified*	Romieu et al. [96]	Children	107	NS	Assessment of biomarkers of traffic-related pollution exposure in EBC and nasal lavage	Higher MDA levels in EBC correlates with greater exposure to traffic-related pollutants; MDA levels are inversely related to FVC and FEV1 and directly related to IL-8 in nasal lavage	HPLC
***Phenotype Not Specified***							
*Stable*	Bartoli et al. [97]	Adults	198 (14)	On medication with ICS (*n* = 41); not on medication (*n* = 23)	Evaluation of MDA levels in EBC in different pulmonary diseases	↑ MDA levels (*p* < 0.001 in A vs. CS); ↑ MDA levels (*p* < 0.05 in UnTxA vs. TxA);	HPLC

↑ = increased; ↓ = decreased; MDA = malondialdehyde; SA = severe asthmatic patients; MMA = mild–moderate asthmatic patients; A = asthmatic patients; CS = control subjects; TxA = treated asthmatic patients; AR = allergic rhinitis patients; GSH = glutathione; MoA = moderate asthmatic patients; TxAE = treated asthma exacerbation patients; AE = asthma exacerbation patients; FEV1 = forced expiratory volume in the first second; FVC = forced vital capacity; IL-8 = interleukin-8; UnTxA = untreated asthmatic patients; TxA = treated asthmatic patients; HPLC = high-performance liquid chromatography.

**Table 3:** Features of studies assessing MDA in individuals affected by urticaria.

URTICARIA								
*Disease*	*Sample*	*Authors*	*Population*	*Cohort (Controls)*	*Treatment Regimens*	*Outcomes*	*Findings*	*Methods of MDA Detection*
***Chronic Urticaria***								
	*Blood*	Galiniak et al. [100]	All	33 (14)	Not on medication	Comparison of markers of oxidative stress	↑ S-MDA levels (*p* < 0.05 in CIU vs. CS)	Spectrophotometry
	*Blood*	Kasperka-Zajac et al. [101]	Adults	64 (19)	Not on medication	Determination of oxidative/antioxidative stress status in CIU patients in the presence or absence of positive response to autologous serum skin test (ASST)	No differences in S- and E-MDA levels, S-CuZn/SOD and S-Mn/SOD activities and E-CuZn/SOD, E-GSH/Px and E-CAT activities	Spectrophotometry
	*Blood*	Sagdic et al. [102]	Adults	61 (36)	Not on medication	Assessment of the role of the oxidative stress in CIU patients	No differences in E-MDA levels; ↑ E-CuZn/SOD (*p* < 0.001 in CIU vs. CS)	Spectrophotometry
	*Blood*	Rajappa et al. [103]	Adults	90 (45)	Not on medication	Determination of platelet oxidative stress andsystemic inflammatory markers	↑ P-MDA and ↓ P-SOD and P-GPx levels (*p* < 0.0001 in CIU vs. CS); positive correlation between USS and P-MDA levels (*p* = 0.001)	HPLC
	*Skin*	Raho et al. [104]	Adults	26 (10)	Not on medication	Evaluation of oxidative stress involvement in CIU	↑ MDA levels and SOD and GSH activities (*p* < 0.005 in CIUASS vs. CIUNASS and CSSS)	Spectrophotometry
***Acute Urticaria***								
	*Blood*	Verma et al. [105]	All	66 (33)	Not on medication	Characterization of oxidative stress status in CADR patients	↑ S-MDA and ↓ S-GSH levels (*p* < 0.001 in CADRs vs. CS)	Spectrophotometry
	*Blood*	Kalkan et al. [106]	Adults	80 (30)	Not on medication	Evaluation of oxidative stress status in AU patients and its clinical significance	↑ S-MDA (*p* < 0.001 in AU vs. CS)	Spectrophotometry
	*Blood*	Kasperka-Zajac et al. [107]	Adults	31 (19)	Not on medication	Evaluation of oxidant/antioxidant profile in NSAID-induced urticaria patients	No differences in S- and E-MDA levels	Spectrophotometry

↑ = increased; ↓ = decreased; the suffixes “E-“, “S-“ and “P-“ refer to erythrocyte, serum and platelet evaluations, respectively; CIU = chronic idiopathic urticaria patients; AU = acute urticaria patients; CS = control subjects; MDA = malondialdehyde; CADR = cutaneous adverse drug reaction patients; USS = urticaria severity score; CIUASS = chronic idiopathic urticaria affected skin sample; CINUASS = chronic idiopathic urticaria non-affected skin sample; CSSS = control subject skin sample; SOD = superoxide dismutase; CuZn/SOD = copper-zinc superoxide dismutase; Mn/SOD = manganese superoxide dismutase; CAT = catalase; GSH/Px = glutathione peroxidase; GSH = reduced glutathione; GPx = platelet glutathione peroxidase; HPLC = high-performance liquid chromatography.

**Table 4:** Features of studies assessing MDA in individuals affected by atopic dermatitis.

ATOPIC DERMATITIS							
*Sample*	*Authors*	*Population*	*Cohort (Controls)*	*Treatment Regimens*	*Outcomes*	*Findings*	*Methods of MDA Detection*
*Blood*	Amin et al. [109]	All	130 (65)	Not on medication	Determination of the extent of lipid peroxidation and antioxidants status	↑ S-MDA levels (*p* < 0.001 in AD vs. CS); ↓ Vit. A, Vit. C (*p* < 0.05 in AD vs. CS) and Vit. E levels (*p* < 0.001 in AD vs. CS)	Spectrophotometry
*Blood*	Galiniak et al. [100]	All	35 (14)	Not on medication	Comparison of markers of oxidative stress	No differences in S-MDA levels	Spectrophotometry
*Blood*	Chung et al. [110]	Children	384 (260)	Not on medication	Assessment of genetic polymorphisms on the risk of AD, and biomarkers analysis	High S-MDA levels in AD. vs. CS (not statistically significant) and ↓ TAC (*p* < 0.001 in AD vs. CS)	Spectrophotometry
*Blood*	Uysal et al. [111]	Children	140 (67)	Not on medication	Evaluation of oxidant/antioxidant balance and its correlation with AD severity	No differences in S-MDA levels; positive correlation between MDA/melatonin ratio and SCORAD index in AD vs. CS	ELISA
*Blood and urine*	Hanusch et al. [112]	Children	53	NS	Oxidant/antioxidant imbalance and determination of MDA levels	No differences in U- and S-MDA levels	GC-MS
*Urine*	Nakai et al. [113]	Adults	70 (20)	On medication with topical corticosteroid and vitamin D3 analogues	Determination of MDA levels	No differences in U-MDA levels; Positive correlation between U-MDA and EASI score (*p* < 0.05)	NS
*EBC*	Peroni et al. [114]	Children	56 (23)	NS	Assessment of inflammatory markers in EBC	High MDA levels in CS vs. AD. (not statistically significant)	LC-MS-MS

↑ = increased; ↓ = decreased; the suffix “U-“ refers to urine, while the suffix “S-“ refers to serum evaluations; MDA = malondialdehyde; AD = atopic dermatitis patients; CS = control subjects; EBC = exhaled breath condensate; TAC = total antioxidant capacity; SCORAD = scoring atopic dermatitis; EASI = Eczema Area and Severity Index; GC-MS = gas chromatography–mass spectrometry; LC-MS-MS = liquid chromatography–tandem mass spectrometry.

## Data Availability

Data sharing not applicable. No new data were created or analyzed in this study. Data sharing is not applicable to this article.
